# Editorial: Glycoconjugates in vaccines and immunotherapeutics

**DOI:** 10.3389/fimmu.2022.941474

**Published:** 2022-08-04

**Authors:** Sachin Shivatare, Kumar Sanjiv, Raghavendra Kikkeri, Roberto Adamo

**Affiliations:** ^1^ Department of Chemistry, The Scripps Research Institute, La Jolla, CA, United States; ^2^ Karolinska Institutet, Science for Life Laboratory, Solna, Sweden; ^3^ Department of Chemistry, Indian Institute of Science Education and Research, Pune, India; ^4^ Research Centre, GlaxoSmithKline (GSK), Siena, Italy

**Keywords:** glycoconjugates, vaccines, infectious diseases, cancer, adjuvants

Carbohydrates are a major, yet often underestimated and poorly explored component of biological systems. Carbohydrates are abundantly expressed on the surface of both eukaryotic and prokaryotic cells, often linked to other biomolecules, such as proteins or lipids, which are referred to as glycoconjugates. Glycoconjugates include a variety of carbohydrate containing molecules, such as proteoglycans, glycoproteins, glycopeptides, glycosides, glycolipids and lipopolysaccharides (1).

Glycoconjugates are involved in in numerous cellular functions. Post-translational glycosylation is essential for structural integrity and stability of proteins and is also involved in signaling, attachment and shielding. Many pathogenic bacteria or viruses displays a diverse array of carbohydrates, which in some cases are involved in entry and even in evasion from host immune system.

Carbohydrates are per se scarcely immunogenic as they are T cell independent antigens: on the opposite, glycoconjugates obtained from covalent linkage of glycans to proteins have been proven to be tremendous tools to prevent diseases such as meningitidis, pneumoniae and salmonellosis (2).

The remarkable differences in glycosylation profiles between healthy and malignant cells plays a critical role also in tumor development and progression (3, 4). Aberrant tumor glycosylation allows to differentiate carbohydrates primarily expressed on cancer cells, the so called tumor-associated carbohydrates. Tumor-associated carbohydrates are known to promote cancer progression by affecting tumor growth, cell invasiveness and negatively regulate immune responses *via* signaling through glycan-binding receptors on immune cells. Therefore, cancer associated glycans are targeted to develop preventive or treatment options such as vaccines and antibodies.

Finally immunomodulatory properties of glycolipids and ability to trigger innate immune responses has been targeted to develop adjuvants used in licensed vaccines and a number of natural and synthetic carbohydrates have been recently designed with this purpose (5).

This Research Topic aims at presenting an overview of glycoconjugates and their potential use as vaccine and therapeutics.

Micoli et al. explores the use of genetically modified outer membrane vesicles (GMMA) as nanosized self-adjuvanted carriers to deliver structurally diverse polysaccharides from different pathogens (including, *Neisseria meningitidis* serogroup A and C, *Haemophilus influenzae* type b, and *Streptococcus* Group A Carbohydrate and *Salmonella* Typhi Vi). Low polysaccharide loading is shown to preserve the immunogenicity of GMMA as antigen and tailored conjugation is proven critical to generate multicomponent vaccines combining different polysaccharides and protein antigens in a single glycoconjugate. In this work GMMA showcase as a versatile platform for conjugation with potential to be exploited in the design of vaccines to combat a variety of infective diseases.

Vaccines will become in the near future a powerful tools to reduce antibiotic use and combat emerging antimicrobial resistance. *Klebsiella pneumoniae* is a major nosocomial pathogen associated and increasing resistance to broad-spectrum β-lactams and carbapenem used for its treatment is worrisome. Lin et al. exploit phage depolymerases to generate glycoconjugates from K1 and K2 capsular polysaccharides which elicited in mice model bactericidal antibodies. A bivalent K1/K2 conjugate vaccine is also demonstrated to protect mice from *K. pneumoniae* infection by the respective capsular type.

Resistance to current treatment option is emerging also for mycotic infections. Boniche-Alfaro et al. characterize a monoclonal antibody (mAbF1.4) raised against the β-glucan rich cell wall of *Paracoccidioides* spp. and investigate in an animal model its efficacy combined with trimethoprim-sulfamethoxazole (TMP/SMX) for the treatment of this fungal infection.

Switching focus to cancer, Barchi gives and overview of nanoparticle carriers to improve the delivery of cancer associated glycans to specific organs and cell types based on tumor-selective approaches. This technology can provide significant support to checkpoint inhibitors, Chimeric Antigen Receptor T-cells, oncolytic virus therapy, monoclonal antibodies, vaccines and all diverse approaches that can contribute to fight cancer from different angles.

Availability of cancer glycans is an important mean to explore use for vaccine application, particularly due to the high heterogeneity of isolated carbohydrates. Phang and Lin report on a novel synthetic route to Type-I and Type-II LacNAc are Gal-GlcNAc disaccharides, bearing a β-(1,3)- or β-(1,4)-linkage respectively, which constitute the backbones of Lewis antigens and are highly expressed in several cancers.

Fuentes et al. utilize a synthetic approaches to obtain well defined analogues of saponin QS21, which is a component of AS01 adjuvant. This offers a chemical route which is alternative to current vegetable extraction to source this class of adjuvant and provides structures to decipher the mechanism of action of this potent adjuvant.

Along with adjuvant, cell targeting is a strategy that can improve the immunogenicity of vaccines. Rentzsch et al. discover that N-tosyl glycan can act as a new class of Langerin receptor ligands that can aid harnessing Langerhans cells engagement for the induction of immune responses and offer a novel way to deliver highly effective vaccines with minimally invasive administration.

This Research Topic can only provide some snapshots of this exciting field but will hopefully attract the readers to dive further into this area.

## Author contributions

RA wrote the manuscript which was revised by all authors. All authors contributed to the article and approved the submitted version.

## Conflict of interest

RA is an employee of GSK.

The remaining authors declare that the research was conducted in the absence of any commercial or financial relationships that could be construed as a potential conflict of interest.

## Publisher’s note

All claims expressed in this article are solely those of the authors and do not necessarily represent those of their affiliated organizations, or those of the publisher, the editors and the reviewers. Any product that may be evaluated in this article, or claim that may be made by its manufacturer, is not guaranteed or endorsed by the publisher.
